# Early response competition over the motor cortex underlies proactive control of error correction

**DOI:** 10.1038/s41598-022-12928-5

**Published:** 2022-06-02

**Authors:** Borja Rodríguez-Herreros, Julià L. Amengual, Jimena Lucrecia Vázquez-Anguiano, Silvio Ionta, Carlo Miniussi, Toni Cunillera

**Affiliations:** 1 Service des Troubles du Spectre de l’Autisme et Apparentés, Centre Hospitalier Universitaire Vaudois, 1011 Lausanne, Switzerland; 2 Sensory-Motor Lab, Department of Ophthalmology, University of Lausanne/Fondation Asile des Aveugles, 1002 Lausanne, Switzerland; 3 Institut des Sciences Cognitives Marc Jeannerod, CNRS UMR 5229, Université Claude Bernard, 69675 Bron, France; 4 Department of Cognition, Development and Educational Psychology, University of Barcelona, 08035 Barcelona, Spain; 5 Center for Mind/Brain Sciences CIMeC, University of Trento, Rovereto, TN Italy; 6 Institute of Neurosciences (UBNeuro), University of Barcelona, Barcelona, Spain

**Keywords:** Cognitive control, Motor cortex

## Abstract

Response inhibition is a fundamental brain function that must be flexible enough to incorporate proactive goal-directed demands, along with reactive, automatic and well consolidated behaviors. However, whether proactive inhibitory processes can be explained by response competition, rather than by active top-down inhibitory control, remains still unclear. Using a modified version of the Eriksen flanker task, we examined the behavioral and electrophysiological correlates elicited by manipulating the degree of inhibitory control in a task that involved the fast amendment of errors. We observed that restraining or encouraging the correction of errors did not affect the behavioral and neural correlates associated to reactive inhibition. We rather found that an early, sustained and bilateral activation, of both the correct and the incorrect response, was required for an effective proactive inhibitory control. Selective unilateral patterns of response preparation were instead associated with defective response suppression. Our results provide behavioral and electrophysiological evidence of a simultaneous dual pre-activation of two motor commands, likely underlying a global operating mechanism suggesting competition or lateral inhibition to govern the amendment of errors. These findings are consistent with the response inhibitory processes already observed in speed-accuracy tradeoff studies, and hint at a decisive role of early response competition to determine the success of multiple-choice action selection.

## Introduction

Human adaptive behavior relies as much on taking suitable actions as on suppressing inappropriate responses. The latter—inhibitory—processes involve both ‘reactive’ and ‘proactive’ control mechanisms^1^. Reactive inhibition (e.g., pedestrians stopping at a red light) is associated to external stimuli and elicits automatic responses. Differently, proactive inhibition (e.g., halt the desire to eat in order to meet weight management goals) actively maintains abstract goal-related cues and operates with endogenous preparatory mechanisms^2,3^. Clear distinction between the underlying neural underpinnings sustaining reactive and proactive inhibitory processes has proven difficult due to limited behavioral paradigms, not ideally suited to isolate one from the other^4,5^.

There is cumulative evidence challenging response inhibition as a classically considered ‘unitary’ brain function^6,7^. Broadly, having foreknowledge of which response must be suppressed has been associated with selective but also slower effortful proactive inhibitory mechanisms, whereas a reflexive and reactive global process prevails when the priority is to stop quickly^8,9^. The dissociation between these two mechanisms of response inhibition resembles the central hypothesis of the dual mechanism of cognitive control (DMC) framework, which postulates that cognitive control operates via two distinct control modes with different temporal dynamics^10^. Specifically, DMC account posits that variability in cognitive control arises from a transient activation of the lateral prefrontal cortex (PFC) when reactive control acts as a ‘late-correction’ mechanism depending on the detection of cognitively demanding events, and a sustained activation of lateral PFC when proactive control relies upon their anticipation and prevention. Nevertheless, neuroimaging studies have exhibited substantial overlapping of the brain networks underlying reactive and proactive inhibitory processes, including areas such as the right inferior frontal cortex (rIFC), the striatum and the subthalamic nucleus^11–14^.

Inspired by previous electrophysiological evidence showing different inhibitory mechanisms when stopping or changing a planned response^15,16^, we sought to experimentally manipulate the degree of inhibitory control within a paradigm of constrained correction of errors. We used a ‘stop-change paradigm’ in which a switch of the direction of the stimulus elicited not only the suppression but also the immediate execution of a fixed alternative response. We manipulated the degree of inhibitory control by either forbidding or encouraging participants to correct their erroneous responses. Therefore, we were able to contrast the natural—automatic—tendency to correct errors against the non-natural condition of refraining the correction^17,18^, resembling the modulation of speed-accuracy tradeoff (SAT) studies^19,20^. In this sense, experimental research in SAT studies has provided insights on the interaction between motor activation and inhibition functions. It is well known that efficient response to a dynamic environment requires an appropriate balance of speed and accuracy, and instructing participants to emphasize speed over accuracy has been shown to modulate not only preliminary response activation, but also inhibitory strength^21^. Whether these inhibitory processes can be explained by response competition involving lateral inhibition, rather than by active top-down inhibitory control, remains still unclear. Some evidence has pointed to the latter^22^, although mutual inhibition between motor areas has also gained substantial support from electromyographic data^23^, showing that transcranial stimulation over the lateral part of the motor cortex of one hemisphere can suppress the excitability of the contralateral motor cortex^24^.

Using event-related potentials (ERPs) and brain oscillatory activity, we investigated the time-course of error- and response-related ERPs as well as two candidate ERPs for successful response inhibition, the N2 and the P3 components. We also measured oscillatory theta- and beta-frequency bands, the recruitment of motor preparatory activity and the onset of the corrective response, indexed with the lateralized readiness potential (LRP)^25^. Because error correction is one of the fastest cognitive processes^26,27^, it seems plausible that corrective responses could proceed in parallel with the impulsive, fast errors that they follow^28^. Some studies have provided direct behavioral and electrophysiological evidence that apparent corrections take place because correct and incorrect responses may sometimes be executed in parallel^29,30^. This would explain why error corrections are often involuntarily made and cannot be consciously withheld^18^. Under such a high degree of response competition, we expected the instruction to inhibit error corrections to elicit the pre-activation of two motor responses, which would be inherently associated to successful response suppression. Otherwise, an active top-down inhibitory control governing successful error correction would entail an enhanced and sustained inhibitory process that should be reflected in the N2-P3 electrophysiological components as well as in the behavioral correlates of response inhibition.

## Materials and methods

### Participants

Nineteen healthy volunteers (12 females; mean age = 20.7 years, SD = 2.46 years) participated in the experiment. We performed a power analysis using G*Power^31^ which determined that a sample of 19 individuals was sufficient to assess the effect of three within-subjects variables with a power of 90.14% and an a priori statistical threshold of 0.05, for a moderate effect size estimate (*η*_*p*_^2^ = 0.06). All the participants had normal or corrected-to-normal visual acuity and no history of neurological or psychiatric disorders. All participants were naïve with respect to the experimental procedures and the hypothesis of the study. Prior to their inclusion in the study, all participants provided written informed consent. The study was approved in Spain by the Bioethics Comission of the University of Barcelona (lnstitutional Review Board IRB00003099) according to the Declaration of Helsinki. All participants were right-handed, as assessed by the Edinburgh handedness questionnaire^32^, and were paid or received extra course credits after completing the experiment.

### Stimuli and procedure

Participants sat in front of a table positioned 45–50 cm below their eyes. Visual stimuli were presented using Presentation® software (v.0.52, Neurobehavioral Systems, Inc., Berkeley, CA, https://www.neurobs.com), running on Windows XP-32SP3 in an Intel Core-i3 computer, and displayed on a 21″ Philips Brilliance 202P4 CRT monitor with a refresh rate of 144 Hz and a resolution of 1024 × 768 pixels. We employed a modified version of the Eriksen flanker task^33^. A horizontal array of five green arrows (4 × 10 cm) was presented for 400 ms in the center of the screen at a Euclidian distance of 60 cm and with a visual angle of ~ 3.4°. Participants were instructed to respond to the direction of the central arrow by using the right index finger when the arrow pointed rightwards, and the left index finger when the arrow pointed leftwards. The four surrounding arrows were either congruent (*Compatible*) or incongruent (*Incompatible*) with respect to the direction of the central arrow. The proportion of compatible and incompatible trials was set to 40/60%. The stimulus onset asynchrony (SOA) was randomly established between 1000 and 1200 ms.

**Figure 1:**
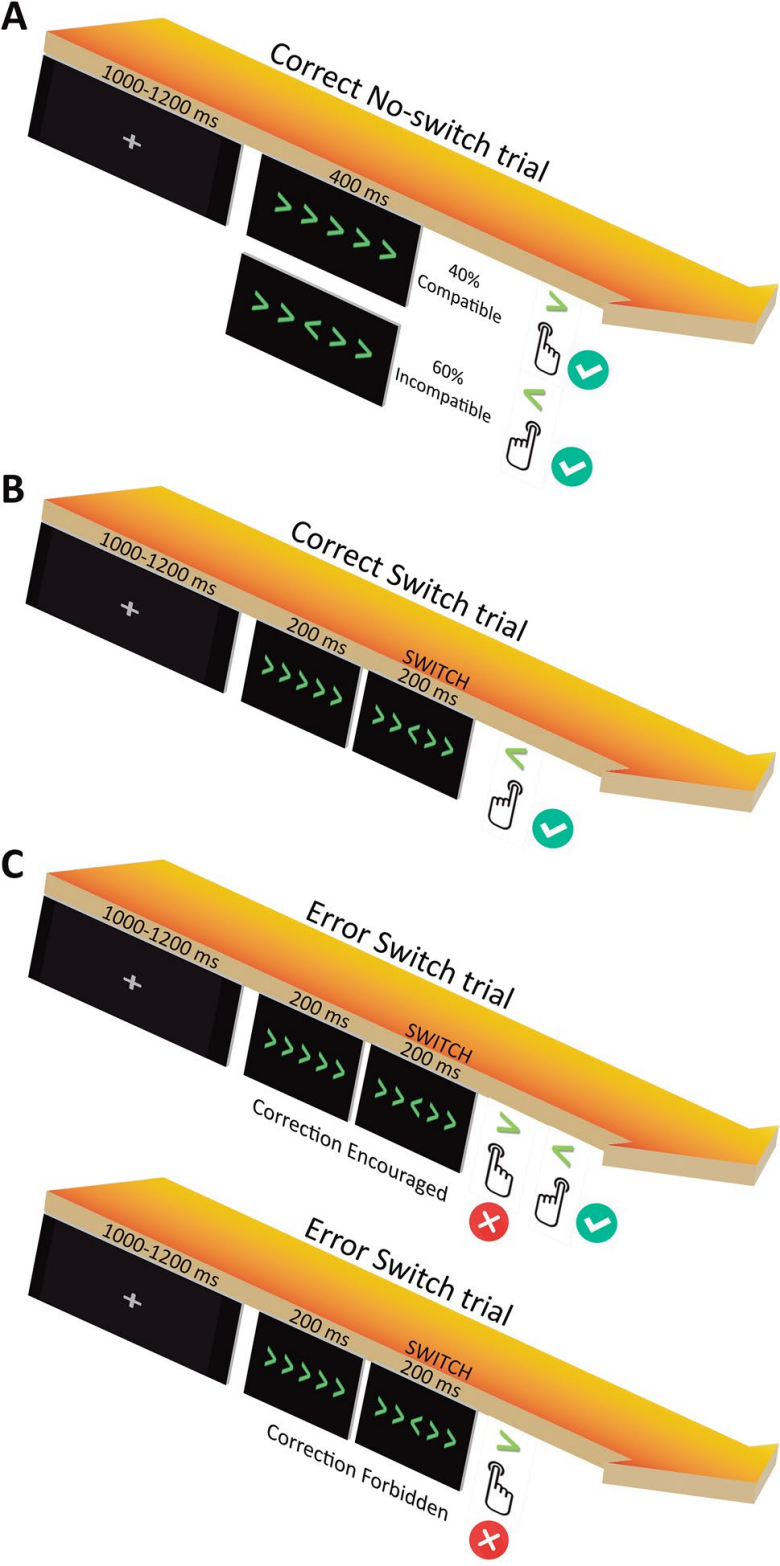
Schematic illustration of the task and experimental design. Participants were asked in all trials to respond to the direction of the central arrow using the corresponding index finger. The experimental session consisted of two blocks, one for each correction instruction. Participants performed 1440 trials in each of these blocks, divided in six runs of 240 trials. Each run had a proportion of 25/75% trials for switch and no-switch trials, respectively. In other words, we had 60 switch trials—presented randomly—per run and a total of 360 switch trials for each correction instruction. With the average of around 50% correct responses to switch, each participant performed approximately 180 correct and 180 erroneous switch trials for each correction instruction. (**A**) Examples of correct responses to compatible and incompatible trials (**B**) Example of a correct response to a switch trial. (**C**) Examples of erroneous responses to a switch trial both in the Correction Encouraged and the Correction Forbidden conditions.

The novel and relevant aspect that differentiates our task from the classical Eriksen version^33^ is that a switch in the direction of the central arrow—equally distributed between compatible and incompatible conditions—was introduced in 25% of trials (Fig. 1). In these switch trials, participants had to avoid responding to the initial direction of the central arrow and respond instead in accordance with its direction after the switch. The onset of the switch (hereafter switch-signal delay, *SwSD*), defined as the time between the go stimulus and the switch signal, was initially set to 200 ms and adapted dynamically on a trial-by-trial basis by means of a staircase-tracking algorithm to compensate for differences between participants^34,35^. The step size, that is, the difference in the SwSD from one switch trial to the other was ± 20 ms. Put simply, after a correct response in a switch trial, the SwSD was increased by 20 ms. In contrast, the SwSD was reduced by 20 ms after an error, thereby modulating, respectively, the difficulty in the next switch trial. Dynamic tracking procedure ensured an overall ratio of p(*response|switch*) of 0.5 in each participant, regardless of their baseline performance and task instructions.

### Experimental design

The experiment consisted of two sessions. The first session was designed as a training block of 15 min to practice the standard flanker task without switch trials (i.e., participants were instructed to respond to the direction of the central arrow with the corresponding index finger). The second session took place 10 days after the training and corresponded to the real experiment with the modified ‘flanker-switch’ paradigm. The task always consisted in responding to the pointing direction (left or right) of the central arrow embedded in the array of 5 identical arrows. However, participants performed this second session divided in two blocks under two different sets of instructions. In one block participants were asked to correct their responses in case they committed an error (*Correction Encouraged* condition), whereas in the other block participants were instructed to inhibit any corrective counteracting response after an error (*Correction Forbidden* condition). These instructions applied for both switch and no-switch trials. A correct response was defined as a response congruent with respect to the direction of the central arrow, and vice versa for erroneous responses. In switch trials, an error took place when the participant did not respond congruently with respect to the direction of the switch. In other words, a correct response to switch was a response that corresponded to the direction of the central arrow after the switch. When correction was encouraged, erroneous responses to switch were followed by a second correct response. The order of the two blocks was counterbalanced among participants. Each experimental block consisted of six consecutive runs of 240 trials each, resulting in a total of 2880 trials for the whole session. Before starting a block, participants performed 20 practice trials to get familiar with the task. The total duration of the experiment was approximately 3 h. To mitigate fatigue effects, a short break of 10 s was included every 40 trials to allow participants to have a short pause. At the end of each of the six runs a longer pause was also enabled until the participant pressed a button to continue with the task. Finally, participants were allowed to have a few minutes break in between the two blocks.

### Behavioral data analysis

We registered the response in each trial and measured the response time (RT). In no-switch trials, RT was the time between the trial onset (i.e., the display of the arrows) and the finger tapping, whereas in switch trials RT was defined as the time between the switch onset and the response. Because the proportions of correct responses were not normally distributed (Shapiro–Wilk test, *p* < 0.008), a two-sided Wilcoxon matched-pairs test was performed to compare the percentage of correct responses, both in switch and no-switch trials, for each correction instruction. The evolution across time of the SwSD was used as an indicator of the inhibitory performance (i.e., SwSD would increase with successful response inhibition and vice versa). To measure the latency of the inhibitory process, we computed the switch-signal RT (SwSRT) following the integration method^36,37^. SwSRT was estimated by subtracting the averaged SwSD from the *n*th RT separately for each condition, where *n* is obtained by multiplying the number of no-switch RTs in the distribution by the overall p(respond/stop-signal). We conducted a 2 × 2 repeated measures analysis of variance (ANOVA) with factors Correction instruction (Encouraged, Forbidden) and Congruency (Compatible, Incompatible) to determine the influence of the two degrees of inhibitory control on the compatibility effect. We also compared RTs between erroneous and correct responses in a 3 × 2 repeated measures ANOVA with factors Correction instruction (Encouraged, Forbidden), Switch (No-Switch trial, Switch trial) and Performance (Correct, Error). The SwSD, the SwSRT, as well as the RT following a previous switch error, were entered into separate 2 × 2 ANOVAs with Correction instruction (Encouraged, Forbidden) and Performance (Correct, Error) as fixed factors. The error rate in switch trials after a previous switch error was also compared between Encouraged and Forbidden correction conditions.

### Electrophysiological recording and analysis

The electroencephalogram (EEG) was recorded with a BrainAmp DC amplifier (*Brain Products GmbH*) from 28 scalp electrodes mounted in an elastic cap (*EasyCap*) in accordance with the standard 10/20 system. The electrodes were located at Fp1/2, F3/4, F7/8, FC1/2, FC5/6, T3/4, C3/4, CP5/6, CP1/2, T5/6, P3/4, O1/2, AFz, Fz, Cz, and Pz). The EEG signal was sampled at 250 Hz and filtered online with a notch-filter (50 Hz) and a high-pass filter (0.016 Hz). The signal was as well referenced online against the right mastoid electrode and re-referenced offline against the half mean of the left mastoid. Electrode impedances were kept below 5 kΩ during the experiment. Eye movements were monitored and recorded by an electrode situated below the right eye. Trials with muscular artifacts and eye blinks were rejected offline, first using a voltage threshold of ± 100 μV and then detecting abrupt voltage changes of ± 25 μV within time windows of 10 ms. The mean rejection rate was 15.9% (± 9.5 SD), comparable between correction instructions (*p* = 0.56). Stimulus-locked (S-Locked) and response-locked (R-Locked) epochs were computed separately from the resulting artifact-free signal for each condition. The length of the epochs was 1000 ms for both modalities (-100 to 900 ms in S-Locked ERPs, and -400 to 600 ms in R-Locked ERPs). The onset of the epochs in the S-Locked ERPs consisted in the presentation of flanker arrays, with the exception of switch trials, in which the onset was determined by the switch cue. Before averaging, the EEG signal was filtered offline with a high-pass filter of 0.1 Hz (2nd order Butterworth filter, 12–40 dB) to remove possible electrode drifts. Finally, but only for illustrative purposes, a lowpass filter of 12 Hz (second order Butterworth filter, 12–40 dB) was applied on the grand average waveforms.

In switch trials, we characterized the error-related ERPs (ERN-Pe compound) and two candidate ERPs commonly reported with successful response inhibition: the frontolateral N2 wave and the frontocentral P3 component. N2 was also considered in correct no-switch trials for the analysis of the flanker incongruency effect. The peak of each component of interest was localized on the grand average waveform (all conditions of interest collapsed). A symmetric time window of 50 or 100 ms was then defined centered on the peak. For each component, mean amplitude measures obtained from the time window were entered into 2 × 2 × 4 repeated-measures ANOVA, with Correction instruction (Encouraged, Forbidden), Performance (Correct, Error) and the four midline electrode locations (AFz, Fz, Cz and Pz) as the studied factors. Specifically, the N2 congruency effect was tested in correct no-switch trials with a 2 × 2 × 4 repeated-measures ANOVA, with Correction instruction (Encouraged, Forbidden), Congruency (Congruent, Incongruent) and the four midline electrode locations (AFz, Fz, Cz and Pz).

In addition to ERPs, time-frequency (TF) analysis was performed by convoluting single-trial data with a complex Morlet wavelet:

$$w\left( {t,f_{0} } \right) = \left( {2\pi \sigma_{t}^{2} } \right)^{ - 1/2 } e^{{\frac{{ - t^{2} }}{{2\sigma_{t}^{2} }}}} e^{{2i\pi f_{0} t}}$$

where the relation f_0_/${\sigma }_{f}$ (where ${\sigma }_{f}$ = 1/($2\pi {\sigma }_{t}$)) was set to 6.7^38^. The frequencies studied ranged from 1 to 40 Hz, with a linear increase of 1 Hz. The time-varying energy—defined as the square of the convolution between wavelet and signal—was computed for each trial and averaged separately for each participant. TF contents were averaged switch-signal locked epochs. Mann-Wilcoxon sum tests were performed for all frequencies and time points to test for significant differences in power between the two degrees of inhibitory control. Percentage of increase/decrease in power for these conditions was entered into the analyses of variance with Correction instruction (Encouraged, Forbidden) and Electrode (AFz, Fz, Cz, Pz) as factors. Significance threshold was set at p < 0.01 and only significant clusters larger than 100 ms were considered. Greenhouse-Geisser epsilon correction was applied in all ERP and TF analyses^39^.

### Lateralized readiness potential (LRP) and current source density analysis (CSD)

We measured LRPs to quantify the motor preparatory activity elicited with the two correction instructions. LRPs were assessed using C3 and C4 electrodes, in which the amplitude of the readiness potential is maximum^40^. The LRP is computed by a double subtraction as shown in the following equation: LRP = left hand (C4–C3) minus right hand (C4–C3). Left and right hands refer to the expected correct hand, and (C4–C3) is the difference in electrical potential between these electrodes^25,41^. The resulting LRP component is negative if participants produce correct responses and positive when they produce a response with the alternative hand, such as when correcting an error. For statistical analysis, mean amplitude values within a 100±50 ms time window around the peak of the motor preparatory activity—located in the grand average waveform separately for correct and error switch trials—were introduced in a 2 × 2 repeated-measures ANOVA with Correction instruction (Encouraged, Forbidden) and Performance (Correct, Error) as the studied factors. All LRP data was filtered with a 12 Hz low-pass filter for the statistical analysis. Huynh-Feldt epsilon correction was applied when necessary.

We used current source density (CSD)—a reference-free technique that computes the second spatial derivative (Laplacian) of the scalp electric potential—, to obtain a more precise quantitative measure of contra- and ipsi-lateral activity in the motor cortex^42^. Laplacian removes the noncortical-induced volume conduction to improve the spatial resolution and provides the location, direction and intensity of the radial current flow that determines an ERP topography^43,44^. We obtained surface Laplacian computed on individual ERP data from C3 and C4 electrodes—positioned bilaterally over the motor cortex—, using the MATLAB-based CSD toolbox^45^ with EEGLAB^46^. We therefore transformed all the averaged ERP waveforms into reference-free CSD estimates (μV/cm^2^ units, head radius = 10 cm). The interpolation was computed using the spherical spline surface Laplacian with computation parameters (50 iterations; spline flexibility *m* = 4; smoothing constant λ = 10^−5^) previously established for our 28-channel recording montage. We collapsed CSD estimates across all conditions, but separately with respect to hemispheric laterality to create contralateral and ipsilateral responses. In other words, the contralateral condition averaged CSD estimates at C3 from right-hand responses with CSD estimates at C4 from left-hand responses, and vice versa for the ipsilateral condition. We filtered the S-locked CSD estimates with a second order Infinite Impulse Response (IIR) Butterworth low-pass filter (12–40 dB) at 20 Hz and entered them into a 2 × 2 repeated-measures ANOVA with Correction instruction (Encouraged, Forbidden) and Performance (Correct, Error) as factors. Based on previous studies on inhibitory control^42^, we measured the area under the CSD waveform within a predefined time window, as well as the slope of the CSD deflection in that window to obtain a second measure not affected by the selected baseline. The slopes were computed by fitting a linear regression to the signal in the time window of interest. Huynh-Feldt and Greenhouse-Geisser epsilon corrections were applied when necessary.

## Results

### Behavioral results

Participants responded correctly in approximately half of the switch trials when correction was forbidden (50.8 ± 3%), indicating an optimal implementation of the staircase-tracking algorithm. When the correction was encouraged, participants were also able to generate correct responses in comparable proportions (48.7 ± 4.4%, *p* = 0.11). Accuracy in no-switch trials reached similar levels (*p* = 0.52) in both the Encouraged (94.6 ± 4.3%) and the Forbidden (92.9 ± 5.9%) correction conditions. Unintended corrections when correction was forbidden—as well as incomplete corrections when correction was encouraged—, were negligible (below 4.5%) in all participants, indicating their proficiency at following the task instructions.

**Figure 2:**
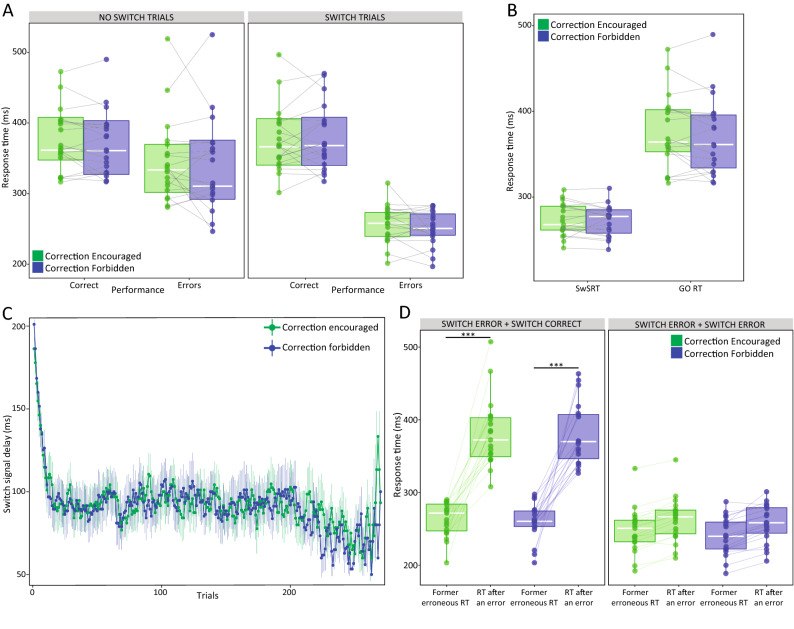
(**A**) Average RT for correct and erroneous responses separately for no-switch and switch trials in the two degrees of inhibitory control. (**B**) Average SwSRT and Go RT in the Encouraged and Forbidden correction condition. (**C**) Changes in the SwSD for each correction instruction are illustrated with respect to trial number. Solid thick lines connect 240 points corresponding to the inter-subject mean for each trial, while the error bars represent the standard error of the mean (SEM). (**D**) Average RT for correct and erroneous responses following an error in switch trials, separately for the two correction instructions. In all boxplots, the bold white line shows the median, and the bottom and top of the box show the 25th (quartile 1 [Q1]) and the 75th (quartile 3 [Q3]) percentile, respectively. The upper whisker ends at highest observed data value within the span from Q3 to Q3 + 1.5 times the interquartile range (Q3–Q1), and lower whisker ends at lowest observed data value within the span for Q1 to Q1-(1.5 * interquartile range). Points not reached by the whiskers are outliers. Significant post hoc group comparisons are represented by solid lines above. *p < .05; **p < .01; ***p < .001.

Figure 2 and Table 1 show the main behavioral results. Complementary behavioral results, such as the Compatibility effect, are described in Supplementary Results (Fig. S1). Figure 2A displays RTs in switch and no-switch trials as a function of the performance and the correction instruction. On average, erroneous responses (298 ms) exhibited shorter RTs than correct (375 ms) responses (Performance: *F*_1,18_ = 184.9, *p* = 6.6e-11, *η*_*p*_^2^ = 0.91). Nevertheless, we observed that this decrease in RT was four times larger in switch trials than in no-switch trials (123 vs. 31 ms; Switch × Performance: *F*_1,18_ = 36.9, *p* = 9.7e-6, *η*_*p*_^2^ = 0.67). The SwSRT (273 vs. 272 ms) and the Go RT (376 vs. 371 ms) were practically identical in the Encouraged and the Forbidden correction conditions (*p* > 0.28 for all comparisons, Fig. 2B). No fatigue effect was found in Go RTs (Block: F_5,90_ = 0.9; *p* = 0.49). The progression of the average SwSD across time for each correction instruction is displayed in Fig. 2C. We observed a rapid and significant decrease of the SwSD in the first trials down to an average slightly below 100 ms (*F*_238,3893_ = 5.85, *p* = 3.4e-48, *η*_*p*_^2^ = 0.88), remaining consistently at this value across 200 trials. We found the same pattern with both correction instructions (*F*_238,3728_ = 0.81, *p* = 0.98).

**Table 1:** Summarized mean values for all behavioral parameters analyzed in the study.

	Correction Encouraged	Correction Forbidden	*p*-value
**No-switch trials**			
Accuracy (%)	94.6 (4.3)	92.9 (5.9)	0.52
RT			
Correct (ms)	376.8 (72)	373.2 (74)	0.76
Error (ms)	320.1 (68)	316.3 (74)	0.75
**Switch trials**			
Accuracy (%)	48.7 (4.4)	50.8 (3)	0.11
RT			
Correct (ms)	373.1 (79)	380.7 (82)	0.86
Error (ms)	257.1 (68)	253.7 (63)	0.52
SwSD (ms)	95.3 (54)	95.1 (57)	0.98
SwRT (ms)	273.1 (19)	272.4 (18)	0.91

We report mean and SD separated for the Encouraged and Forbidden correction conditions.

We also evaluated the performance of each participant after the commission of an error in a switch trial. After a switch error, we observed that the RT of the next switch trial was on average 67 ms longer (*F*_1,18_ = 127.2, *p* = 1.4e-9, *η*_*p*_^2^ = 0.88). Figure 2D reveals that this RT slowing down was clearly dependent on the performance of that subsequent switch trial (*F*_1,18_ = 68.1, *p* = 1.6e-7, *η*_*p*_^2^ = 0.79): An additional erroneous response exhibited an RT only 16 ms larger than that of the former error (244 vs. 260 ms, *t*_(18)_ = -2.4, *p* > 0.05 after correction for multiple comparisons); whereas a correct response to switch showed an RT 120 ms larger than that of the preceding switch error (260 vs. 381 ms, *t*_(18)_ = -13.5, *p* = 7e-16, d = 2.19). These differences were indicative of a ‘switch cost’ in correct responses to switch, alike when error correction was encouraged or forbidden.

### EEG results

Analogously to the behavioral results, complementary EEG results are described in Supplementary Results, such as the Compatibility effect (Fig. S2A) and the error-related potentials (Fig. S2B).

#### Inhibitory processing: N2 and P3 components

**Figure 3:**
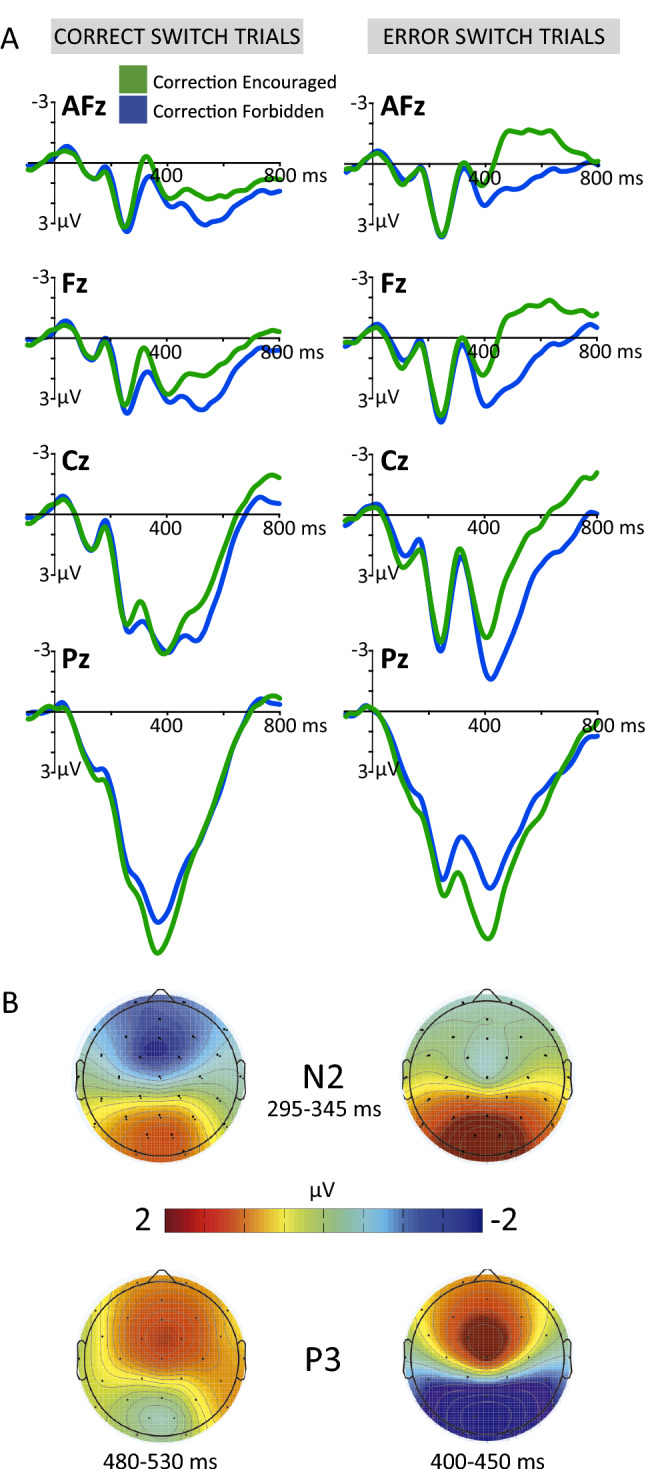
(**A**) S-locked grand average from midline electrodes for correct (left) and error (right) switch trials, separately for the Encouraged (green) and Forbidden (blue) correction conditions. (**B**) Topographical mapping of N2 and the P3 components.

Figure 3A shows the modulation of the S-locked N2 component as a function of performance and correction instructions. Within the 295-345 ms time window, N2 amplitude was larger in erroneous compared to correct responses (Performance: *F*_1,18_ = 15.88, *p* = 8.6e-4, *η*_*p*_^2^ = 0.47). Furthermore, the instructions given to the participants also affected N2 amplitude (Correction instruction x Electrode: *F*_3,54_ = 16.11, *p* = 5.8e-5, *η*_*p*_^2^ = 0.47). This interaction was driven by a larger N2 amplitude for correct responses in frontal electrodes when error amendments were encouraged (AFz: *t*_(18)_ = -2.39, *p* = 0.03, *d* = -0.28; Fz: *t*_(18)_ = -2.34, *p* = 0.03, *d* = -0.43), as shown in Fig. 3B.

The analysis of the modulation of the P3 component revealed a different pattern when compared to the preceding N2. We found a significant Correction instruction x Electrode interaction (*F*_3,54_ = 23.96, *p* = 1.2e-5, *η*_*p*_^2^ = 0.57), driven by a significantly larger P3 amplitude in posterior regions when participants were instructed to correct their errors (*Pz*: *t*_(18)_ = -3.06, *p* = 0.007, *d* = -0.4, Fig. 3B). The second positive phasic deflection following the P3 component—peaking at 525 ms in anterior electrodes—was also considered for the analysis. We observed that this deflection was significantly larger when participants were instructed to correct their errors (*F*_1,18_ = 8.65, *p* = 0.009, *η*_*p*_^2^ = 0.33), and overall, in error switch trials (*F*_1,18_ = 10.88, *p* = 0.004, *η*_*p*_^2^ = 0.38).

#### Time-frequency results

We found an increase of the theta activity peaking around 400 ms after the switch onset in the two correction conditions. The increase was larger in central locations (t_(18)_ > 4.8, *p* < 0.001 for all comparisons). Mu (12–15 Hz) and beta (20–30 Hz) activity were desynchronized in central and posterior locations between 200 and 800 ms after the switch (*F*_1,18_ > 3.4, *p* < 0.05 for both correct and erroneous responses). The statistical maps across frequencies and time did not exhibit any significant TF cluster when comparing the two degrees of inhibitory control in correct responses (Fig. S3). In contrast, we found that beta synchronization was larger in error switch trials when correction was forbidden, approximately 800 ms after the switch, and prominently in anterior regions (Fig. S4).

#### LRP and CSD results

**Figure 4:**
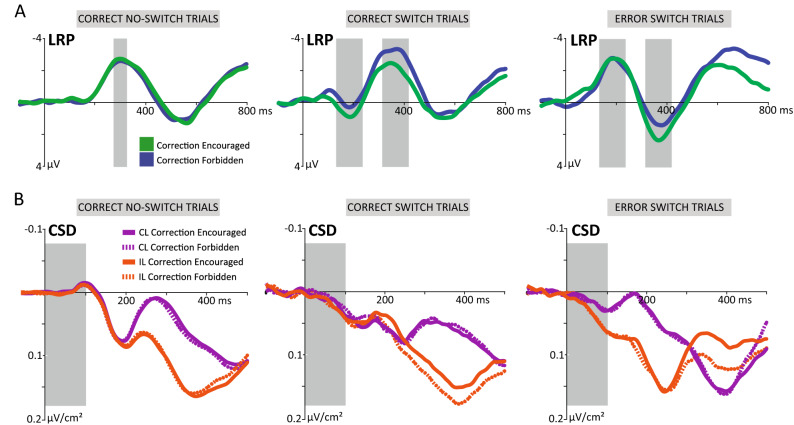
(**A**) S-locked LRPs in correct no-switch, correct switch and error switch trials for Encouraged and Forbidden correction conditions. The gray boxes indicate the analyzed time windows associated to the response preparation and, if required, to the processing of the switch. (**B**) Grand average S-locked CSD waveforms of the contralateral (purple) and ipsilateral (orange) motor preparatory activity separately for correct no-switch, correct switch and error switch trials. Solid lines represented the Encouraged correction condition whereas dashed lines correspond to trials in which the correction had to be withheld. *IL, ipsilateral; CL, contralateral.*

We measured the motor preparatory activity separately in correct (1st peak at 184 ms; 2nd peak at 360 ms) and error switch trials (1st peak at 184 ms; 2nd peak at 368 ms), as well as in correct no-switch trials (peak at 300 ms). As expected, the LRP in the first time window (134–234 ms) displayed a remarkable polarization with opposite sign associated to the performance in switch trials (*F*_1,18_ = 228.34, *p* = 1.1e-11, *η*_*p*_^2^ = 0.93; Fig. 4A). Before a correct response to the switch, we however found a similar pattern of the motor preparatory activity when corrections were encouraged or forbidden (*F*_1,18_ = 1.47 *p* = 0.24). On average, the motor preparatory activity encapsulated in the second time window (correct: 310–410 ms, error: 316–416 ms) was conspicuously linked to the response to the switch. The comparison of the LRPs generated with each correction instruction revealed a significantly higher amplitude in the preparation of correct switch responses when the instruction was to refrain any subsequent correction (*F*_1,18_ = 14.67, *p* = 0.001, *η*_*p*_^2^ = 0.45). As expected, motor preparatory activity in this second time window was larger for correct than for erroneous responses (*F*_1,18_ = 63.62, *p* = 2.6e-7, *η*_*p*_^2^ = 0.79), whereas the interaction between performance and correction instruction was again non-significant (*F*_1,18_ = 0.05, *p* = 0.82). Interestingly, motor preparatory activity of erroneous responses to switch in the second time window reflected a marginal decrease when participants were instructed to withhold the correction (*t*_(18)_ = -2.04, *p* = 0.06, *d* = -0.5), likely obeying to the fact that error corrections were not effectively suppressed on time. Finally, we observed comparable motor preparatory activity in no-switch correct responses between the two correction instructions (*t*_(18)_ = 1.01; *p* = 0.32; *d* = 0.14).

The use of CSD estimates allowed us to separately quantify the contralateral and ipsilateral electrophysiological correlates associated to response preparation with each correction instruction. We found a positive deflection of the CSD 100 ms after the onset of the initial stimulus (Fig. 4B). The statistical analyses in the 0–100 ms time window revealed a main effect of hemispheric laterality (*F*_1,18_ = 30.41, *p* = 3.1e-5, *η*_*p*_^2^ = 0.63), and a significant Laterality x Performance interaction (*F*_1,18_ = 11.77, *p* = 0.003, *η*_*p*_^2^ = 0.4). When decomposing the interaction, we observed that correct and erroneous responses to a switch exhibited a similar level of motor preparatory activity in the contralateral hemisphere (*t*_(18)_ = -0.48, *p* = 0.64). Nonetheless, motor preparatory activity in the ipsilateral hemisphere was significantly larger in correct switch trials (*t*_(18)_ = -2.94, *p* = 0.009, *d* = -0.6). These results were consistent under the two degrees of inhibitory control, and suggest that an early and sustained pattern of motor preparatory activity from the two hemispheres, not only the contralateral, would be required before the execution of a successful response to the switch.

To avoid a spurious baseline bias, we analyzed the CSD deflection in the same time window by calculating the mean amplitude at the beginning (0–20 ms) and at the end (74–94 ms) of the slope, and computing a linear regression for each participant and correction instruction. The baseline-free mean amplitude values reproduced the significant main effect of laterality (*F*_1,18_ = 11.09, *p* = 0.004, *η*_*p*_^2^ = 0.38) and a Laterality x Performance interaction (*F*_1,18_ = 4.83, *p* = 0.04, *η*_*p*_^2^ = 0.21). Again, the motor preparatory activity observed in correct and error switch trials only differed in the ipsilateral side (*t*_(18)_ = 2.05, *p* = 0.05, *d* = 0.44).

## Discussion

**Figure 5:**
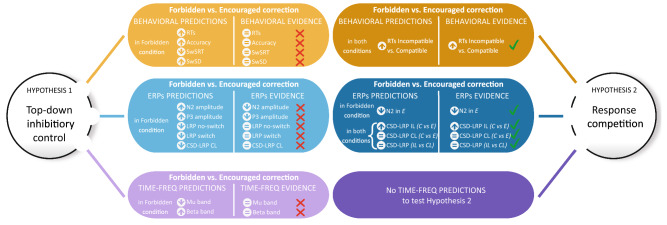
Flowchart of the behavioral (orange), ERPs (blue) and time–frequency (purple) predictions and evidences for both the top-down inhibitory control (pale) and the response competition (dark) hypotheses. Green check marks and red crosses indicate whether data supports or refutes the hypothesis. IL, ipsilateral; CL, contralateral; C, correct; E, errors.

In this study, we examined behavioral and electrophysiological correlates of proactive inhibitory control while participants performed a modified version of the Eriksen flanker task^33^, which allowed us to elicit both automatic and endogenously prepared response suppression. We used a ‘stop-change paradigm’ in which a switch of the direction of the stimulus prompted not only the suppression but also the immediate execution of a fixed alternative response. Our experimental paradigm thus allowed us to create situations in which proactive inhibitory control is at use to cope with overcorrection of errors. Restraining or encouraging the correction of errors did not affect the time course of the behavioral and electrophysiological correlates associated with response inhibition. However, as early as 100 ms after the stimulus onset, we found an early bilateral activation of both the correct and the incorrect response when participants effectively withheld the correction of an error. These findings provide evidence of a global operating mechanism suggesting response competition to govern the amendment of errors. In turn, our results point out that inhibitory mechanisms associated with response inhibition are not necessary to explain sustained top-down control over behavior. Figure 5 depicts a flowchart of the top-down inhibitory control and response competition contrasting models summarizing their respective predictions and the evidence observed in the present study.

Reactive inhibition is typically investigated with tasks including a condition in which a prevalent response tendency needs to be stopped^47^. As a result, the use of the stop signal reaction time (SSRT) as an objective quantitative estimate of the time needed to abort an already-initiated response has been broadly supported^11,48^. On average, SwSRT—the equivalent measure to SSRT in this study—was practically indistinguishable when participants were instructed to correct or to withhold an error just committed. Moreover, the trial-by-trial tracking exhibited a robust similarity of the SwSD under the two degrees of inhibitory control, stabilizing slightly below 100 ms the delay at which a response could still be effectively suppressed. These findings suggest that the internal speed of reactive stopping was not modulated by changes in the degree of inhibitory control. We also observed that initiating correct responses to the switch took significantly longer compared to a previous erroneous trial, reflecting careful response strategies reminiscent of the ‘switch cost’ that underlies task-set reconfiguration processes^49^. Contrarily, two consecutive erroneous responses to the switch showed comparable RTs. Our data thus corroborates previous evidence for flexible trial-by-trial behavioral adaptations after unsuccessful response inhibition^50^.

We provide electrophysiological evidence supporting the validity of the two degrees of inhibitory control that we introduced in the experiment, and rule out possible confounding factors that could mislead the interpretation of the results. One example is the replication, with the two correction instructions, of the well-established conflict-related N2 component. We reproduced the greater amplitude for incongruent—relative to congruent—trials, interpreted as evidence for the visual competition of the flanking arrows to enable the execution of the correct response^51–53^. Besides, the ERN-Pe compound was identified immediately after the commission of an error, as the output of an evaluative system engaged in monitoring motor conflict^30,54^.

The larger N2 amplitude observed in erroneous responses dovetails well with previous reports showing an increase of the N2 component in failed stop trials^55,56^. Interestingly, the fact that the N2 component is not enhanced when correction is forbidden—and thus a second posterior response must be inhibited—casts doubt on the role of this component as an index of response inhibition. One might argue though that the N2 frontal differences reflect an overlap with conflict resolution processes exclusively when a correction is encouraged^15^. Altogether, our results are in agreement with the view that N2 is more likely to reflect attentional processes related to the conflictive stop signal and response competition, rather than the success of an inhibitory process^57,58^.

Regarding the frontocentral P3 waveform, a large body of literature supports its relationship with successful response inhibition^59–62^. Nonetheless, there is still a lack of consensus on the specific neural process that P3 subserves. We observed a larger P3 amplitude in posterior regions when participants were encouraged to correct their errors, which would tentatively reflect the process of suppressing the old response and reprogramming the new action^63^. However, several studies refuse to link the P3 with response inhibition per se, but they rather support its association with a post hoc evaluation of the performance^61,64^. This interpretation is supported by the fact that P3 peaks too late relative to the SSRT^65^, albeit other studies suggested P3 onset latency as a reliable neural marker of response inhibition^66^. Lastly, oscillations in the beta band are known to show a prominent event-related desynchronization during the preparation and execution of manual responses^67,68^. We observed a frontocentral rebound of beta power 800 ms after the switch when participants committed an error and were instructed to withhold the correction. Frontal beta increases have been previously associated to post-error slowing^69^.

LRPs have been extensively used to reflect preferential response activation in the motor cortex. The error-correction mechanism studied here relates to the detection of errors and the intentional release of a corrective motor command using the contralateral limb. Our analysis of the LRPs validated the reverse of their polarity in erroneous responses to switch. Moreover, response preparation in correct trials was inhibited around 150 ms after the stimulus onset, and this pattern was consistent under the two degrees of inhibitory control. This LRP lateralization for the incorrect response up to 150 ms exhibits how far participants pushed their limits without committing an error^16^, and is in line with the short time between the LRP onset and the response when speed was privileged over accuracy in SAT studies^70^. The larger preliminary motor activation observed when error correction was inhibited is expected, since there is no correction mechanism to be implemented and only one response must be produced. These results are also consistent with the SAT studies showing larger LRP amplitudes when subjects were instructed to respond fast^19,20^. Conversely, previous studies had already shown that, when error correction was encouraged, LRPs immediately displayed a shorter lateralization interval^16,30^. Altogether, this evidence reinforces the view that the amplitude of the LRP reflects the balance between response activation and lateral inhibition processes^22^.

Consistent with neurostimulation studies reporting reduced corticospinal excitability 150 ms after a stop signal^71–73^, the CSD estimates reflected bilateral increases of preliminary motor activity after 100 ms in correct responses to switch. This inhibition latency is also coincident with the estimation of around 150 ms from partial response electromyographic data^74^. However, only the contralateral motor cortex exhibited substantial preparatory activity when participants committed an error. It is reasonable to think that these results might reveal a global operating strategy in which two responses would be pre-activated simultaneously in case a response reconfiguration is required due to a switch. Consistently, bilateral pre-activation of both responses would entail an optimal capacity to quickly detect erroneous responses and execute the corrective motor command^30^. Our behavioral data supports this view, by showing that RTs in error switch trials were largely shorter than in error no-switch trials, likely reflecting two different underlying neural processes. According to top-down models of inhibitory control, only hyper-fast responses—roughly below 250 ms—will be more likely to escape inhibition, suggesting that the activation of these responses finished before triggering the correspondent inhibitory processes^27,28^. Our findings thus suggest that early bilateral activation of both the correct and the incorrect response, thus invoking response competition processes to a comparable extent, might be a crucial component of the cascade of processes that ultimately result in an effective response suppression.

Under the premises of the DMC framework, the proactive control mode would require the sustained maintenance of a particular type of information—the goals and rules of a task—for an optimal performance. Preserving an active representation of predefined goal-related cues has been also associated with biasing attention in favor of task-relevant information^3,75^, which in our study could explain the anticipation of the response to the expected upcoming switch signal. To do that, our findings indicate that generating a global—bilateral—pattern of motor activation for both the correct and the incorrect response would be crucial to effectively select the desired sequence of stimulus features over other competing combinations. According to this view, inhibition would occur because of response competition during action selection among conflicting task-relevant representations that correspond to the goals and the rules for achieving a behavior. Our results are consistent with the main tenet of the DMC framework, and suggest that optimizing sustained attentional monitoring would enable an adequate proactive control of response inhibition. They speak to a growing debate in cognitive control research about active mechanisms of response suppression and motivate richer models of how to cancel a prepared or initiated action. Further research is warranted to outline putative common elements of proactive inhibition across other functional domains, such as emotional and motivational impulses.

## Supplementary Information

Supplementary Information.

## Data Availability

The datasets generated during and/or analyzed during the current study are available from the corresponding author on reasonable request.
