# Supplementary material for: Early response competition over the motor cortex underlies proactive control of error correction
Source: Sci Rep. 2022 Jun 2;12:9232. doi: 10.1038/s41598-022-12928-5 (PMC9163130; doi:10.1038/s41598-022-12928-5)
Supplement: Supplementary file 1 — Supplementary Information. [file 41598_2022_12928_MOESM1_ESM.pdf]

Supplementary material

Early response competition over the motor cortex underlies proactive control of error correction

Borja Rodríguez-Herreros[1,2], Julià L. Amengual[3], Jimena Lucrecia Vázquez-Anguiano[4], Silvio Ionta[2], Carlo Miniussi[5] and Toni Cunillera[4*]

Supplementary results

Behavioral. We reproduced a compatibility effect both when encouraging and forbidding error correction (Compatibility: $F_{1,18}$ = 156.1, p = 2.6e-10, η_p^2 = 0.9; **Figure S1A**), therefore demonstrating a correct implementation of the flanker task. RTs in incompatible trials were 21 ms ($t_{(18)}$ = -7.9, p = 1.8e-9, d = 1.28) and 20 ms ($t_{(18)}$ = -6.58, p = 1e-7, d = 1.07) longer when the correction was encouraged and forbidden, respectively. The error rate after a previous switch error was comparable between Correction Encouraged and Correction Forbidden conditions (Correction Instruction: $F_{1,18}$ = 2.68, p = 0.12; **Figure S1B**). Finally, a trial-by-trial analysis of the switch RTs after a switch error shows that, on average, RTs increased quickly after the first trials to a value within the 300-360 ms, being consistent through blocks and between correction instructions (**Figure S1C**).

EEG results. The compatibility effect was reflected on the modulation of the N2 component at central electrodes in no-switch trials (Cz; Compatibility: $F_{1,18}$ = 4.9, p = 0.038, η_p^2 = 0.34). **Figure S2A** shows that the S-locked (*incongruent – congruent*) difference waveform was comparable between the two degrees of inhibitory control (Correction Instruction: $F_{1,18}$ = 3.0, p = 0.1; Correction Instruction x Compatibility: $F_{1,18}$ = 0.06; p = 0.82), indicating that encouraging or forbidding the correction of errors exerted no influence on the processing of congruent and incongruent sets of stimulus arrays.

The neural correlates of the errors committed in switch trials were examined by analyzing the R-locked (*error – correct*) difference waveform on the ERN-Pe compound (**Figure S2B**). While no difference in the modulation of the ERN was found when comparing the two degrees of inhibitory control (Correction Instruction: $F_{1,18}$ = 2.77, p = 0.11; Correction Instruction x Electrode: $F_{3,54}$ = 0.42, p = 0.61), we observed a significantly larger Pe amplitude

when the correction of errors was forbidden ($F_{1,18}$ = 9.31, p = 0.007, η_p^2 = 0.34). The similar modulation of the ERN in both corrective conditions proved that missing to withdraw the shift in the direction of the central arrow was effectively computed as an error.

Remarkably, both the amplitude and the latency of the ERN elicited when error correction was forbidden were virtually identical to that of the Correction Encouraged condition, in which the errors committed were immediately corrected. The larger error-related positivity of the Pe component elicited when error correction was forbidden could be reminiscent of a stronger subjective assessment of the error, likely due to the fact that errors cannot be amended. And vice versa, being aware of the possibility to rectify an error likely reduced error significance, depicting a shorter Pe amplitude [1,2].

Supplementary references

1 Falkenstein, M., Hoormann, J., Christ, S. & Hohnsbein, J. ERP components on reaction errors and their functional significance: a tutorial. *Biol Psychol* **51**, 87-107, doi:10.1016/s0301-0511(99)00031-9 (2000).

2 Falkenstein, M., Hohnsbein, J. & Hoormann, J. Differential processing of motor errors. *Recent advances in event-related brain potential research*, 579-585 (1996).

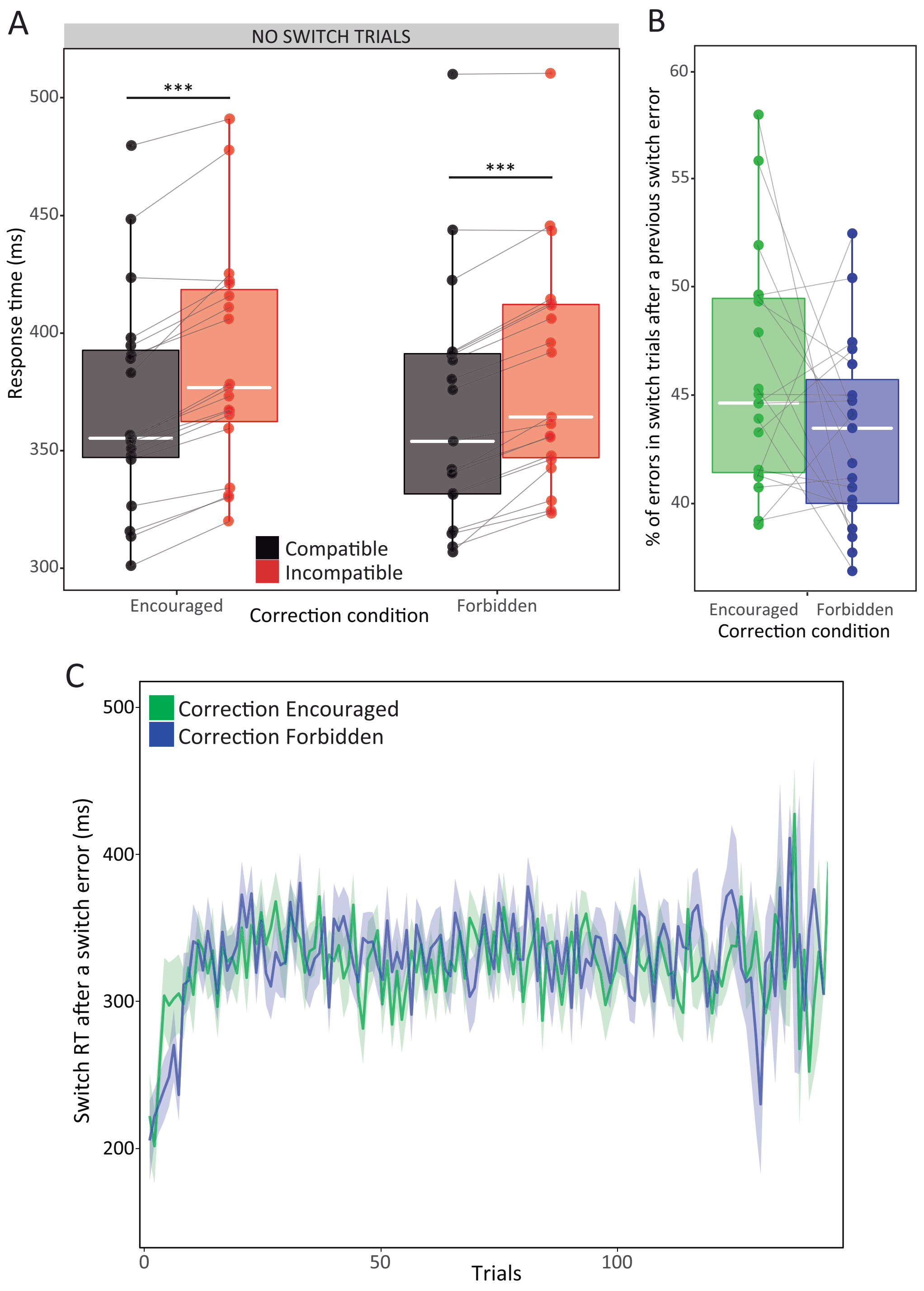

Fig. S1. (A) Average RT for compatible and incompatible no-switch trials in the Encouraged and Forbidden correction condition. **(B)** Proportion of two consecutive erroneous responses to a switch in the Encouraged and Forbidden correction condition. **(C)** Changes in the RT after an error in switch trials for each correction instruction are illustrated with respect to trial number. Shaded areas represent the SEM. In all boxplots, the bold white line shows the median, and the bottom and top of the box show the 25th (quartile 1 [Q1]) and the 75th (quartile 3 [Q3]) percentile, respectively. The upper whisker ends at highest observed data value within the span from Q3 to Q3+1.5 times the interquartile range (Q3–Q1), and lower whisker ends at lowest observed data value within the span for Q1 to Q1-(1.5 * interquartile range). Points not reached by the whiskers are outliers. Significant post hoc group comparisons are represented by solid lines above. *p<.05; **p<.01; ***p<.001.

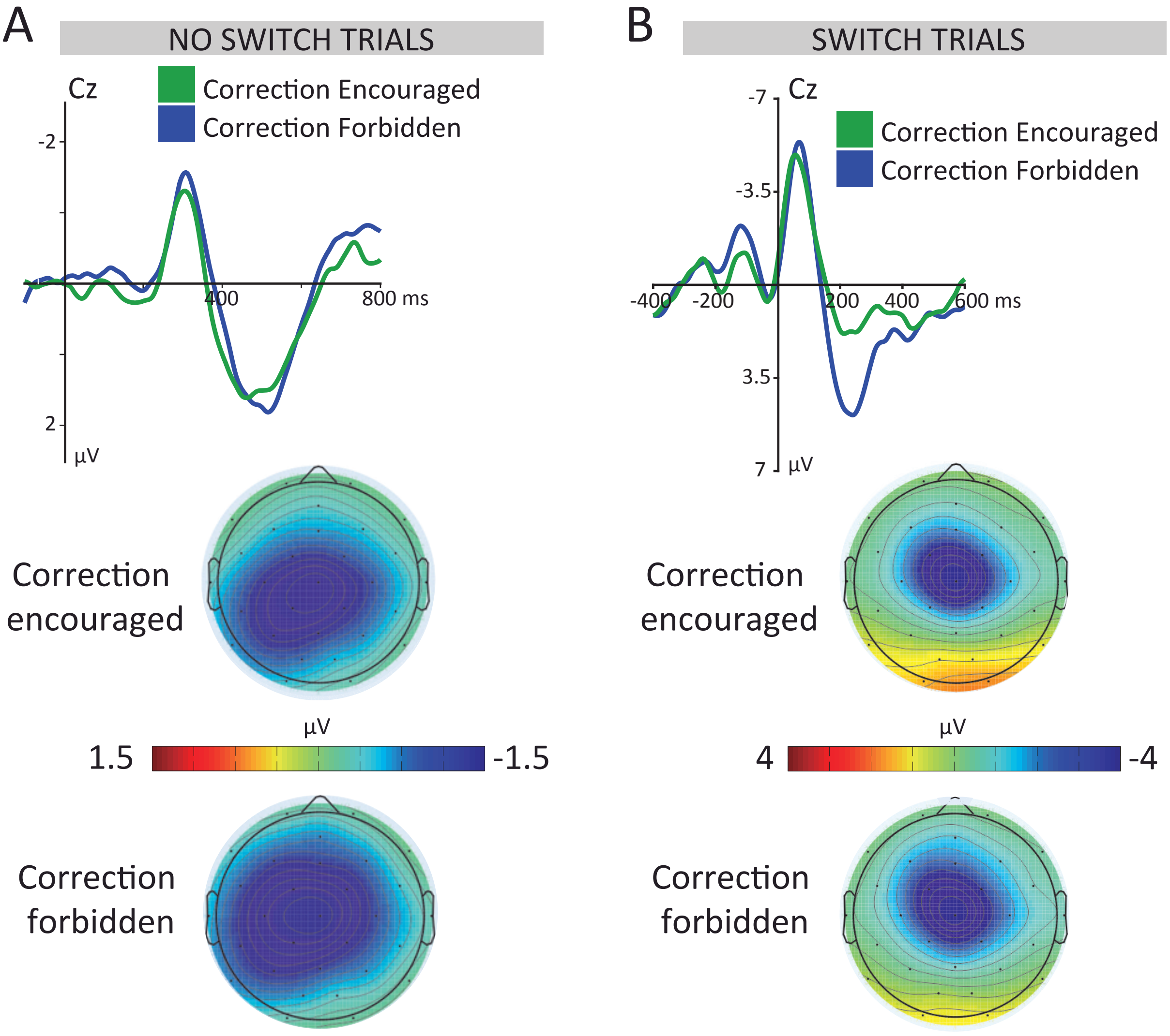

Fig. S2. (A) Grand average Stimulus-locked electrophysiological correlates of the compatibility effect in no-switch trials. Incongruent minus congruent difference waveform in Cz electrode, together with the 2D isovoltage topographical mapping (283-333 ms) illustrating the scalp distribution of the N2 component for each correction condition. **(B)** Response-locked grand average and 2D isovoltage topographical mapping (45-95 ms) of the error *minus* correct difference waveform eliciting an ERN-Pe compound in switch trials for each correction condition. ERPs were filtered with 12 Hz and 20 Hz low-pass filters for R-locked and S-locked epochs, respectively, but only for presentation purposes.

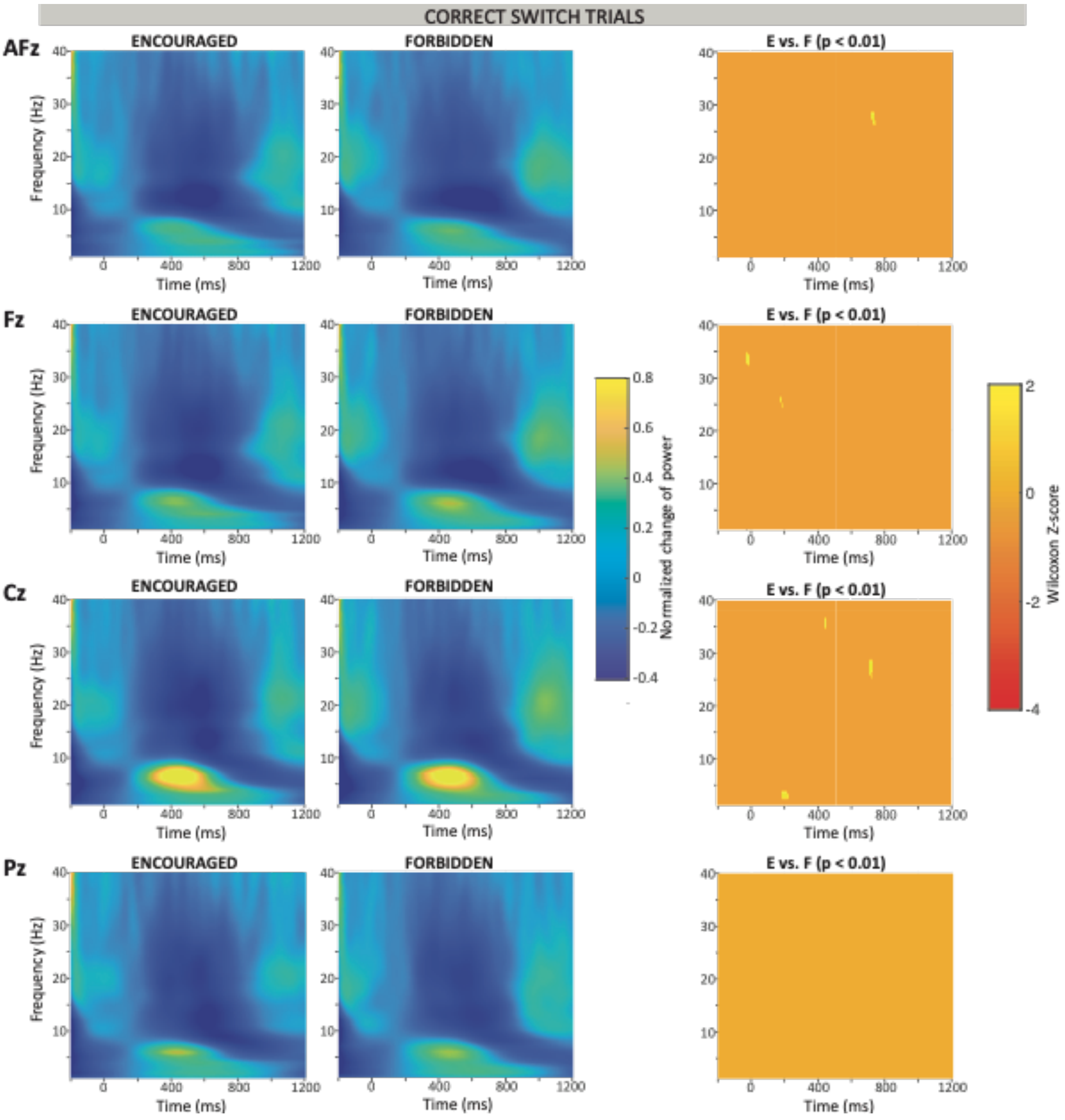

Fig. S3. Switch-locked TF and statistical maps of the event-related oscillatory activity recorded from Afz, Fz, Cz and Pz electrodes from frequencies between 1 to 40 Hz during correct responses to switch for both Encouraged and Forbidden correction conditions. Changes of power relative to baseline (-200 to 0 ms prior to the switch). The right column presents point-by-point Mann-Wilcoxon tests between Encouraged and Forbidden correction conditions. Only significant p-values ($p < .01$) are represented.

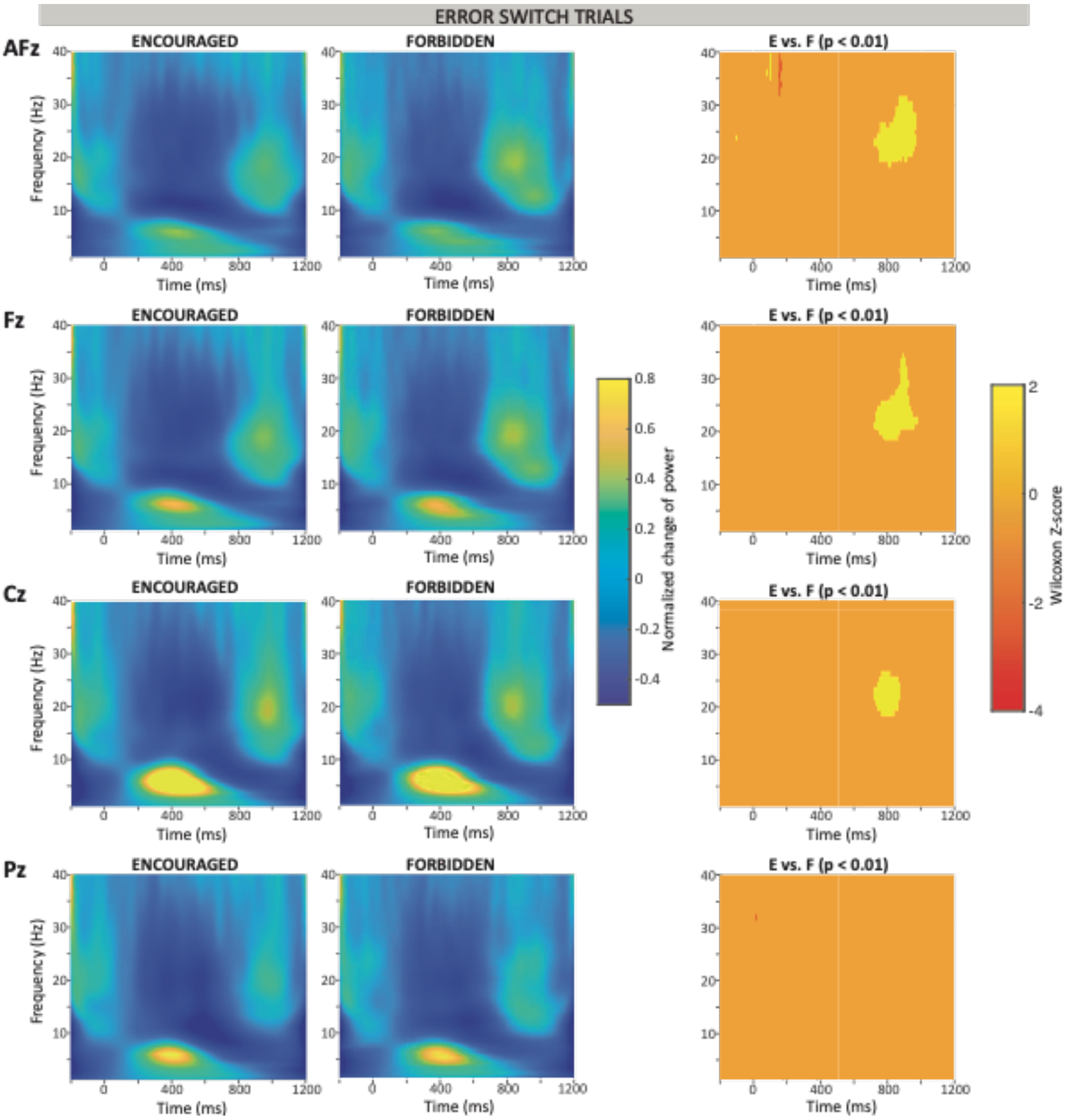

Fig. S4. Switch-locked TF and statistical maps of the event-related oscillatory activity recorded from Afz, Fz, Cz and Pz electrodes from frequencies between 1 to 40 Hz during erroneous responses to switch for both Encouraged and Forbidden correction conditions. Changes of power relative to baseline (-200 to 0 ms prior to the switch). The right column presents point-by-point Mann-Wilcoxon tests between Encouraged and Forbidden correction conditions. Only significant p-values ($p < .01$) are represented. The analysis of the latency onset determined that the beta synchronization after failing to inhibit a correction started 150 ms earlier (850 ms vs. 1000 ms after the switch onset).
